# Editorial: Innate Immune System Guiding Physiological Plasticity in Invertebrates

**DOI:** 10.3389/fphys.2022.947707

**Published:** 2022-06-14

**Authors:** Bharat Bhusan Patnaik, Yong Seok Lee, Yeon Soo Han, Yong Hun Jo

**Affiliations:** ^1^ Department of Biosciences and Biotechnology, Fakir Mohan University, Balasore, India; ^2^ Department of Biology, College of Natural Sciences, Soonchunhyang University, Asan, South Korea; ^3^ Department of Applied Biology, Institute of Environmentally-Friendly Agriculture, College of Agriculture and Life Sciences, Chonnam National University, Gwangju, South Korea

**Keywords:** innate immunity, *Tenebrio molitor*, immune priming, physiological plasticity, host-pathogen interaction

Innate immunity mechanisms have provided an effective means of surveillance and protection against parasites and pathogens in invertebrates. With the lack of adaptive immunity, invertebrates rely on the plasticity conferred by innate immunity to eliminate pathogens, and perhaps more importantly discriminating them from other microorganisms. Moreover, credible evidences from studies in infection immunity, transplantation immunity, individual, and transgenerational immune priming have highlighted the development of immunological memory, a characteristic feature of adaptive immunity, under the innate immune response specialty of invertebrates. This Research Topic gathers different contributions highlighting the phenotypic plasticity in terms of innate immunity traits underlying the physiological status of invertebrates. These contributions expand our understanding of the novel components of innate immunity signaling cascades, elucidation of physiological fitness traits and pathogen evasion mechanisms intricately attributed to invertebrate survival.

The mealworm beetle, *Tenebrio molitor* has provided rich insights to unravel the mysteries of innate immune mechanisms in invertebrates pertinent to host-pathogen interactions. In the last decade or so, many critical components of Toll, Imd, Jak-Stat, and autophagy signaling cascades have been deciphered in the beetle model using elegant biochemical, genetic, and genomics approaches. These studies have collectively highlighted antimicrobial innate immune responses in *T. molitor* while characterizing the conserved pathway components regarding antimicrobial peptide (AMP) production. The first article of this Research Topic (Bae et al.) discusses the anti-Gram-negative function of another Toll ligand spätzle 1b (TmSpz1b) identified from the RNA sequencing database of *T. molitor*. Other studies, reported by the same research group, have critically characterized the function of *T. molitor* spätzle isoforms such as spätzle 5 (TmSpz5) and spätzle 6 (TmSpz6) in regulating AMP production in the Malpighian tubules in response to *Escherichia coli*, and against *E. coli* and *Staphylococcus aureus*, respectively (Edosa et al., 2020; Ali Mohammadie Kojour et al., 2021). TmSpz1b was characterized as a secretory protein with a conserved cystine-knot domain as evidenced in other insects including spätzle protein in *Drosophila melanogaster*. Further, the authors reported the requirement of TmSpz1b in regulation of AMP genes in hemocytes and fat bodies and showing susceptible phenotype against *E. coli* infection. The inclusion of TmSpz1b in regulating Toll pathway was suggested as TmSpz1b silenced individuals showed downregulation of *TmDorsal* (a critical NF-kB factor regulating Toll cascade mechanisms) transcripts. In the second article, Ko et al. functionally characterized the inhibitor of nuclear factor kappa B kinase epsilon isoform from *T. molitor* (TmIKKε), that is a transcriptional regulator of Imd pathway and Janus kinase (JNK) activation and apoptosis (downstream of Imd) pathway mediated by the phosphorylation of DIAP1 as evidenced from studies involving *D. melanogaster* IKKε. Further, they suggested the putative involvement of TmIKKε in conferring immunity against *E. coli* infection. Interestingly, in gut the Toll NF-kB factors *TmDorX1* and *TmDorX2* were downregulated in *TmIKKε*-silenced individuals suggesting systemic immunity in gut mediated by the Toll pathway. However, in an earlier study, *TmIKKγ*-silenced individuals showed less *TmRelish* (NF-kB transcription factor of Imd pathway) transcript abundance highlighting antimicrobial innate immune response mediated by Imd pathway (Ko et al., 2020).

Regarding host-pathogen interactions, another contribution by Wronska et al. has discussed apoptosis (changes in caspase activity) as an integral component of innate immune system particularly in the context of entomopathogenic fungus *Conidiobolus coronatus* infection of greater wax moth, *Galleria mellonella*. Hypothetically, the authors assumed that in immunocompetent insect hemocytes, fungal infection stimulates apoptosis and influences eicosanoid levels, largely synthesized by cyclooxygenase (COXs), lipoxygenase (LOX) as well as the cytochrome P450 pathway. Significant increase in phospholipase A2 (PLA2) activity in the infected larval hemolymph acted as a biomarker for eicosanoid biosynthesis and correlated well to a rise in general activity of caspases and prostaglandins. Hence, immunity in insects under the challenge of fungal infection creates new directions to study the apoptotic phenomenon regulated by not only caspases but also by other factors such as eicosanoids. It would also be interesting to correlate the activation of PLA2 with gene activation of AMPs in Dorsal- and Relish-silenced insect larvae to suggest the requirement of Toll/Imd signal pathways for eicosanoid biosynthesis. It has been reported that the eicosanoid mediating innate immune responses in insects are functionally linked to Toll/Imd signal pathways in the beet armyworm, *Spodoptera exigua* and in the red flour beetle, *Tribolium castaneum* (Shrestha and Kim 2010; Park and Kim 2012), providing compelling evidence for including PLA2 activity and caspase-mediated apoptosis as an efficient immune surveillance mechanism against pathogenic infections in insects.

The physiological capacity to actively metabolize uric acid thus limiting *Plasmodium* parasite survival was addressed in Kwon and Smith, by analyzing both urate oxidase (UO) and allatoicase (ALLC) expression by qRT-PCR and RNAi. The authors suggested that the mosquito host manipulates uric acid metabolism to limit pathogenic infections. This was demonstrated by silencing UO (metabolism of uric acid to urea) in mosquito host, that resulted in increased uric acid levels, enhancing parasite survival. Hence, it was substantiated from the study that integral to vectorial capacity (mosquito vector competence) is the nitrogen metabolism pathway. Earlier studies in *Drosophila* host (under bacterial challenge) and in tsetse flies (under *Trypanosoma brucei* infection) have also supported the hypothesis that uric acid promotes the success of parasitic and pathogenic infections (MacLeod et al., 2007; Lang et al., 2019).

The phenomenon of “immune priming” was evaluated by Hidalgo and Armitage by infecting *D. melanogaster* with one of the two bacterial species, *Lactococcus lactis* or *Providencia burhodogranariea* (either heat-killed or inactivated with formaldehyde). To the surprise of the authors and in contrast to previous reports on immune priming in insects, primary exposure treatments did not provide a survival benefit to pathogen-challenged host suggesting that immune priming is not a ubiquitous phenomenon in insect immunity and may require specific circumstances to occur. The dynamic nature of host resistance over the infection course was also suggested as pre-exposed flies showed resistance that varied over the course of infection. The pre-exposure advantage on a secondary challenge was nullified in the study contemplating future studies to understand adaptive value of this phenomenon considering specific host-pathogen combinations. As discussed in a previous report, the pronounced immune priming phenomenon in industrial insects such as *T. molitor* could be crucial to obtain sustainable stocks of insect population for multiplication as food (Ali Mohammadie Kojour et al., 2022).

We hope that the readers of this Research Topic would use this resource as a useful reference tool while advancing their research directions towards intricacies of innate immune response in host-pathogen interactions.
